# Supplementary material for: Chinese and UK participants' preferences for physical attractiveness and social status in potential mates
Source: R Soc Open Sci. 2019 Nov 20;6(11):181243. doi: 10.1098/rsos.181243 (PMC6894565; doi:10.1098/rsos.181243)
Supplement: Full analysis R code and output [file rsos181243supp1.pdf]

Code ▾

Chinese and UK Participants' Preferences for Physical Attractiveness and Social Status in Potential Mates

Zhang et al.

- Data Processing
  - Load Data
  - Translation Checks
  - Join Data
  - Data Exclusions
  - General Descriptives
  - Visualisations
- Confirmatory Analyses
  - Prediction 1 (Attractiveness)
    - UK Participants
    - CN Participants
    - Descriptives
  - Prediction 2 (Status I)
    - UK Participants
    - CN Participants
    - Descriptives
  - Prediction 3 (Status II)
    - Descriptives
- Exploratory Analyses
  - Creativity
    - UK participants
    - CN participants
    - Descriptives
  - Kindness
    - UK participants
    - CN participants
    - Descriptives
  - Liveliness
    - UK participants
    - CN participants
    - Descriptives
  - Age and Ideal Partner Age
    - UK Participants
    - CN Participants
    - Descriptives
  - Comparision among traits
    - Female mate preference for each trait in short-term relationships

        - Female mate preference for each trait in long-term relationships
        - Male mate preference for each trait in short-term relationships
        - Male mate preference for each trait in long-term relationships
- Text
    - Attractiveness (Prediction 1)
    - Social Status (Prediction 2)
    - Social Status (Prediction 3)
    - Creativity
    - Kindness
    - Liveliness
    - Age

Code

Data Processing

Load Data

Code

Translation Checks

Code

Code

Join Data

Code

Data Exclusions

Ns for Raw Data (pre-exclusions)

Code

sex <fctr>	region <fctr>	att <int>	creat <int>	kind <int>	lively <int>	status <int>
female	CN	153	153	153	153	153
female	UK	127	127	127	127	127
male	CN	172	172	172	172	172
male	UK	132	132	132	132	132

4 rows

Keep only people who:

1. passed the definition check for the relevant word
2. <= 3 SDs from the mean for that sex

3. completed both LT and ST

Excluded due to failing definition check

Code

sex <fctr>	region <fctr>	att <dbl>	creat <dbl>	kind <dbl>	lively <dbl>	status <dbl>
female	CN	29	12	0	9	5
female	UK	23	5	7	39	5
male	CN	25	11	2	10	13
male	UK	17	9	12	39	11

4 rows

Excluded due to not responding to dollars question

Code

sex <fctr>	region <fctr>	att <dbl>	creat <dbl>	kind <dbl>	lively <dbl>	status <dbl>
female	UK	1	1	1	0	1
male	CN	1	1	1	2	1
male	UK	0	0	1	0	1

3 rows

Excluded due to not completing both LT and ST after outlier exclusion

Code

sex <fctr>	region <fctr>	att <dbl>	creat <dbl>	kind <dbl>	lively <dbl>	status <dbl>
female	CN	4	3	3	3	4
female	UK	4	5	5	2	3
male	CN	4	7	10	7	7
male	UK	2	2	2	2	0

4 rows

Code

General Descriptives

Number of post-exclusion participants per region, sex and trait:

Code

sex <fctr>	region <fctr>	att <dbl>	creat <dbl>	kind <dbl>	lively <dbl>	status <dbl>
female	CN	120	138	150	141	144
female	UK	99	116	114	86	118
male	CN	142	153	159	153	151
male	UK	113	121	117	91	120

4 rows

Code

sex <fctr>	region <fctr>	trait <chr>	pre <dbl>	def <dbl>	missing <dbl>	outlier <dbl>	total_excluded <dbl>	post <dbl>
female	CN	att	153	29	0	4	33	120
female	UK	att	127	23	1	4	28	99
male	CN	att	172	25	1	4	30	142
male	UK	att	132	17	0	2	19	113
female	CN	creat	153	12	0	3	15	138
female	UK	creat	127	5	1	5	11	116
male	CN	creat	172	11	1	7	19	153
male	UK	creat	132	9	0	2	11	121
female	CN	kind	153	0	0	3	3	150
female	UK	kind	127	7	1	5	13	114

1-10 of 20 rows Previous **1** 2 Next

Means and SDs per per region, sex and trait (post-exclusions):

Code

sex <fctr>	region <fctr>	te... <fctr>	n <int>	attractiveness <chr>	creativity <chr>	kindness <chr>	liveliness <chr>
female	CN	LT	151	19.52 (10.23)	14.44 (7.48)	25.37 (11.22)	13.08 (7.64)
female	CN	ST	151	33.26 (15.29)	11.50 (8.52)	18.98 (11.23)	14.55 (9.08)
female	UK	LT	122	22.78 (8.56)	14.97 (8.57)	31.46 (10.22)	16.77 (7.11)
female	UK	ST	122	33.64 (15.17)	11.75 (8.38)	25.84 (11.45)	17.09 (7.69)
male	CN	LT	170	27.63 (15.83)	12.73 (6.59)	27.03 (10.9)	17.12 (7.6)
male	CN	ST	170	40.45 (20.86)	9.830 (7.97)	20.35 (11.92)	16.76 (8.23)
male	UK	LT	129	28.90 (11.21)	14.74 (7.21)	27.55 (9.74)	19.53 (6.5)

male	UK	ST	129	42.50 (18.74)	11.34 (8.05)	18.78 (12.29)	20.08 (9.25)

8 rows

Visualisations

Code

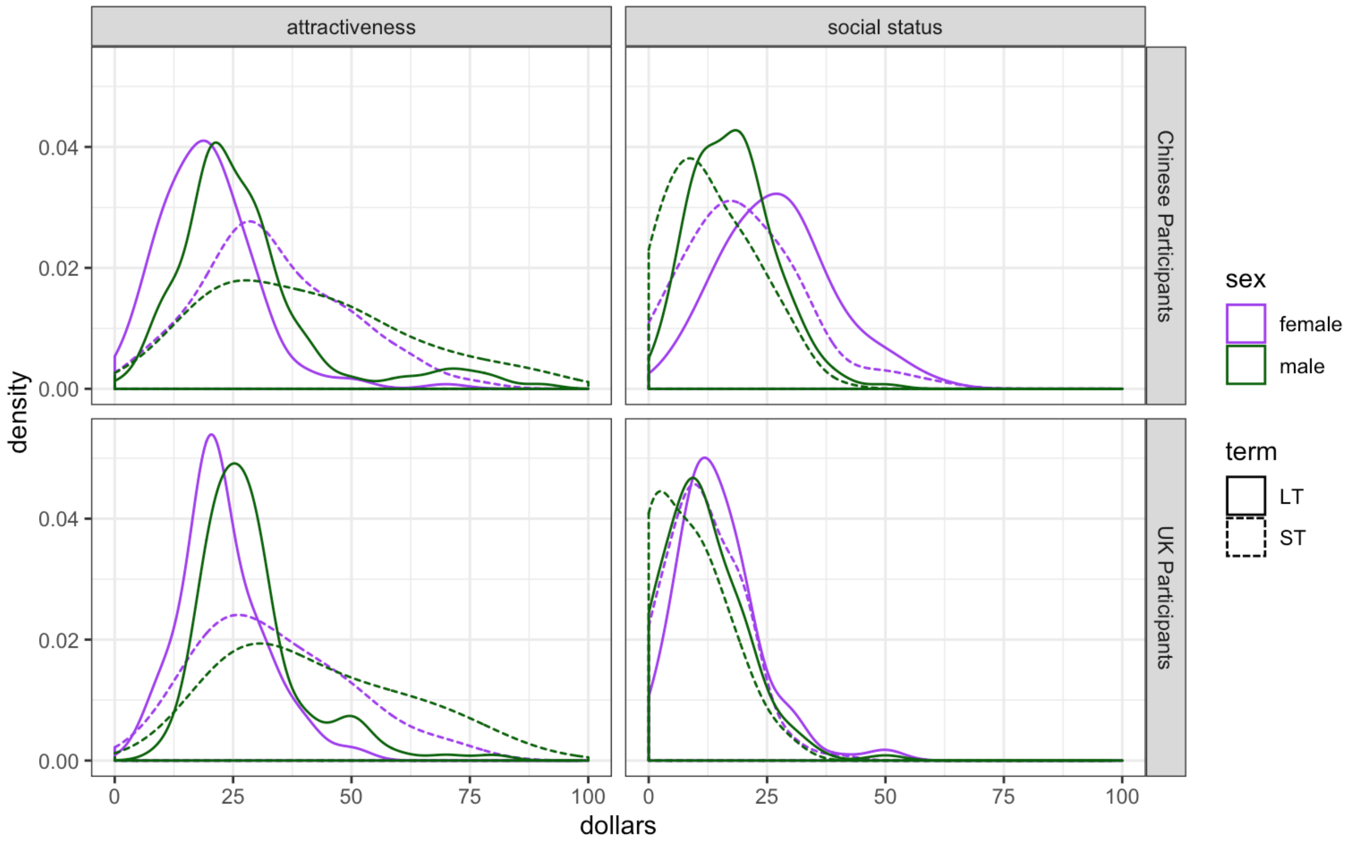

Code

Code

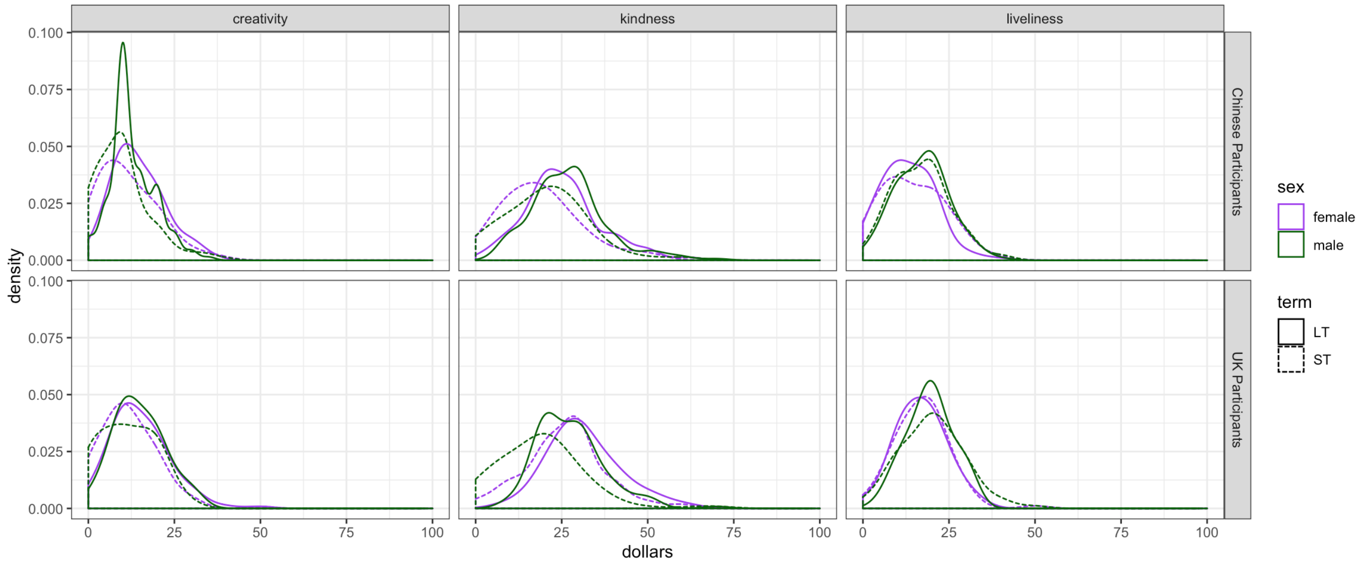

Code

Confirmatory Analyses

Prediction 1 (Attractiveness)

1A. Men will allocate more mate dollars to physical attractiveness than women in both Chinese and UK samples.

1B. This sex difference will be particularly pronounced when choosing for potential short-term partners than for potential long-term partners.

UK Participants

Code

```
## Anova Table (Type 3 tests)
##
## Response: att
##     Effect     df    MSE          F  pes p.value
## 1      sex 1, 210 281.27  21.09 ***  .09  <.0001
## 2     term 1, 210 114.56 137.80 ***  .40  <.0001
## 3 sex:term 1, 210 114.56       1.73 .008     .19
## ---
## Signif. codes:  0 '***' 0.001 '**' 0.01 '*' 0.05 '+' 0.1 ' ' 1
```

CN Participants

Code

```
## Anova Table (Type 3 tests)
##
## Response: att
##     Effect     df    MSE          F   pes p.value
## 1      sex 1, 260 375.43  20.28 ***   .07  <.0001
## 2     term 1, 260 151.27 151.54 ***   .37  <.0001
## 3 sex:term 1, 260 151.27       0.18 .0007     .67
## ---
## Signif. codes:  0 '***' 0.001 '**' 0.01 '*' 0.05 '+' 0.1 ' ' 1
```

Descriptives

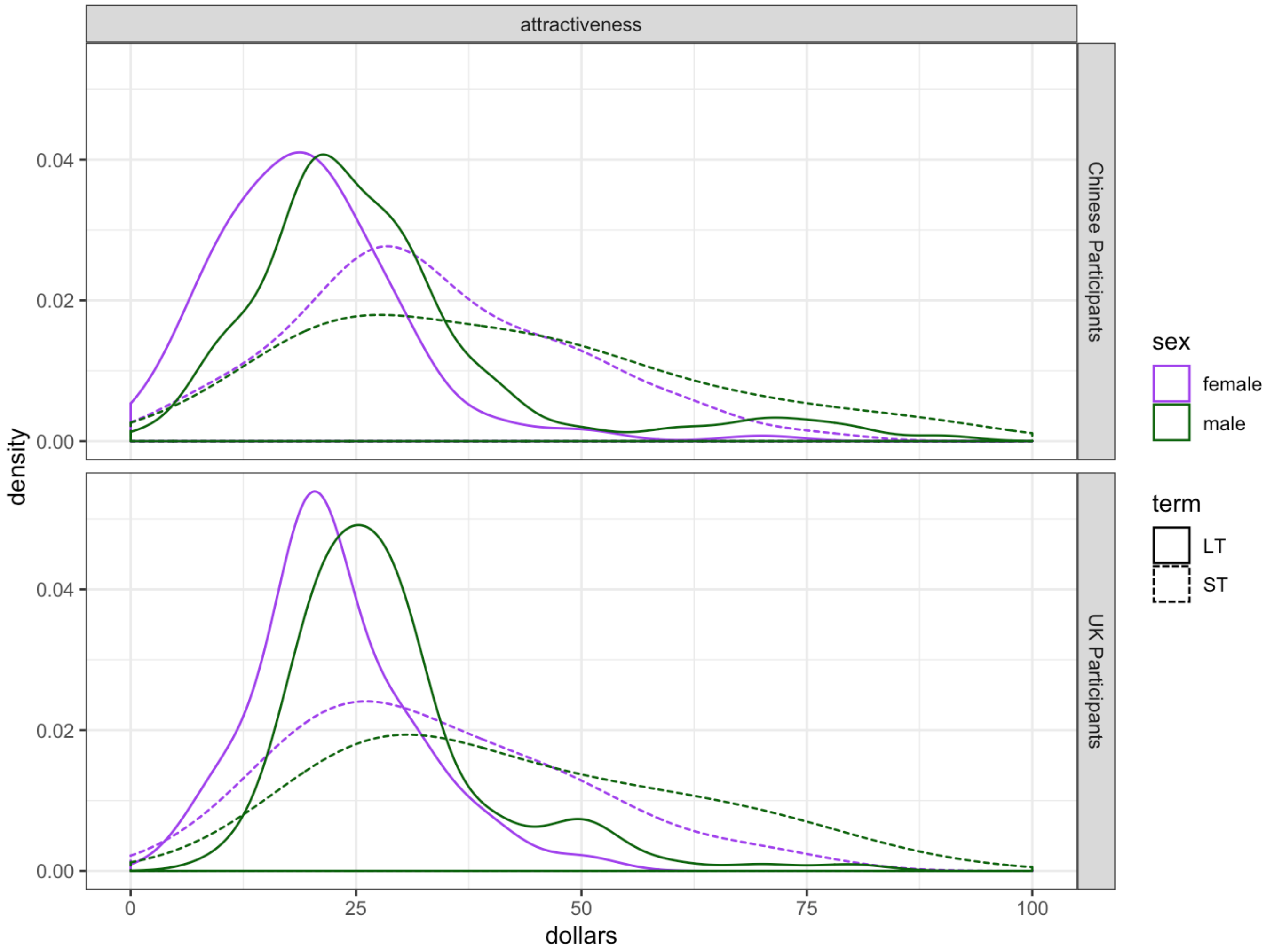

Code

region <fctr>	male <dbl>	female <dbl>	att_sex_dif <dbl>
CN	34.04	26.39	7.65
UK	35.70	28.21	7.50

2 rows

Code

region <fctr>	term <fctr>	male <dbl>	female <dbl>	att_sex_dif <dbl>
CN	LT	27.63	19.52	8.11
CN	ST	40.45	33.26	7.19
UK	LT	28.90	22.78	6.12
UK	ST	42.50	33.64	8.87

4 rows

Prediction 2 (Status I)

2A. Women will allocate significantly more mate dollars to social status than men in both the UK and Chinese samples.

2B. This sex difference will be significantly more pronounced when choosing for potential long-term partners than short-term partners.

UK Participants

Code

```
## Anova Table (Type 3 tests)
##
## Response: status
##     Effect     df    MSE         F    pes p.value
## 1      sex 1, 236 101.35 12.01 ***     .05   .0006
## 2     term 1, 236  36.47 31.11 ***     .12  <.0001
## 3 sex:term 1, 236  36.47      0.01 <.0001     .92
## ---
## Signif. codes:  0 '***' 0.001 '**' 0.01 '*' 0.05 '+' 0.1 ' ' 1
```

CN Participants

Code

```
## Anova Table (Type 3 tests)
##
## Response: status
##     Effect     df    MSE         F pes p.value
## 1      sex 1, 293 146.07 68.63 *** .19  <.0001
## 2     term 1, 293  73.17 74.98 *** .20  <.0001
## 3 sex:term 1, 293  73.17    3.10 + .01     .08
## ---
## Signif. codes:  0 '***' 0.001 '**' 0.01 '*' 0.05 '+' 0.1 ' ' 1
```

Descriptives

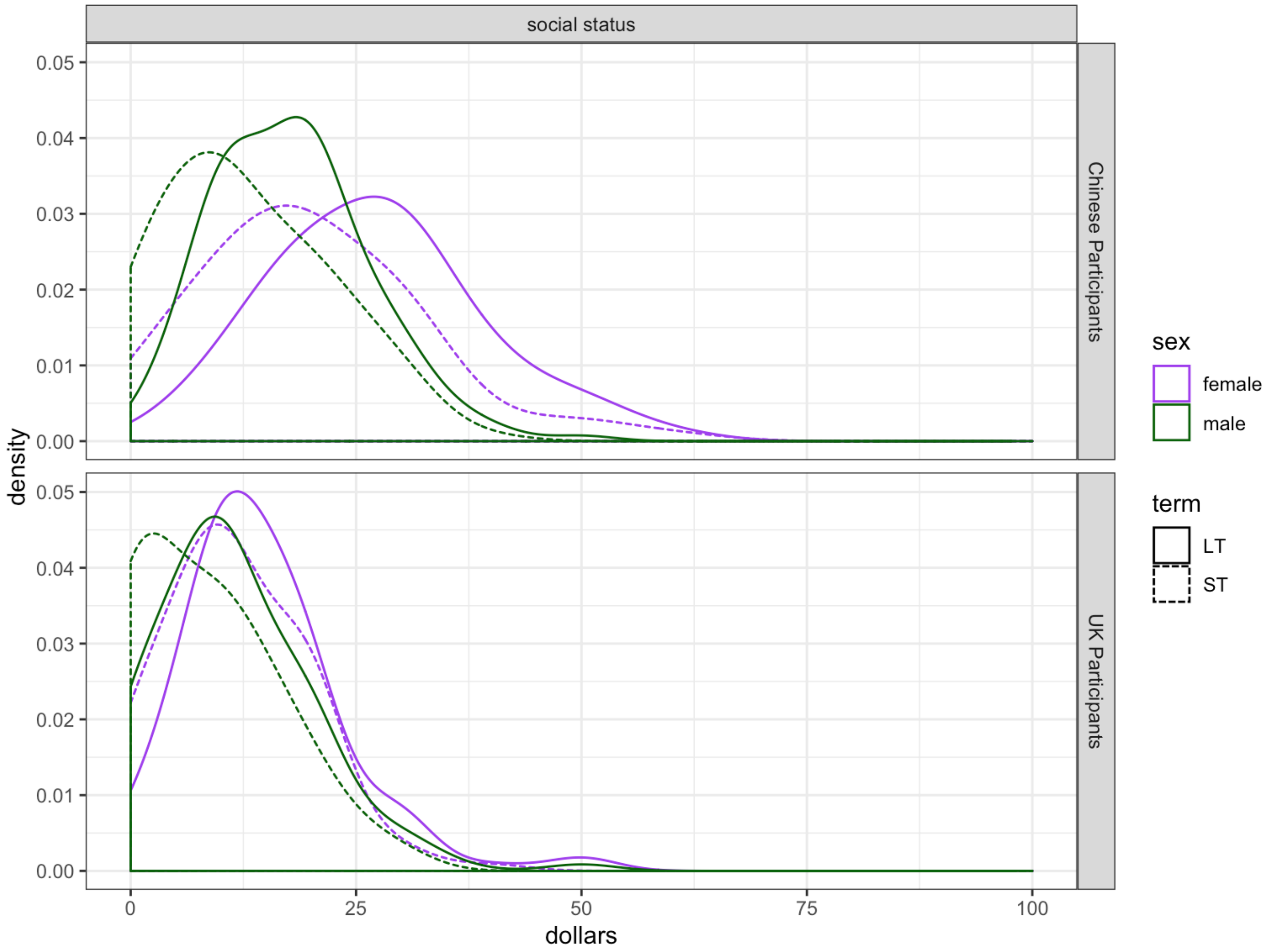

Code

region <fctr>	male <dbl>	female <dbl>	status_sex_dif <dbl>
CN	15.28	23.52	-8.25
UK	9.94	13.14	-3.20

2 rows

Code

region <fctr>	term <fctr>	male <dbl>	female <dbl>	status_sex_dif <dbl>
CN	LT	17.71	27.19	-9.49
CN	ST	12.85	19.85	-7.01
UK	LT	11.51	14.65	-3.14
UK	ST	8.37	11.62	-3.25

4 rows

Prediction 3 (Status II)

Chinese women will allocate significantly more mate dollars to social status than UK women will in the long-term context.

Code

```
## Anova Table (Type 3 tests)
##
## Response: status
##   Effect     df    MSE         F pes p.value
## 1 region 1, 260 109.09 93.52 *** .26  <.0001
## ---
## Signif. codes:  0 '***' 0.001 '**' 0.01 '*' 0.05 '+' 0.1 ' ' 1
```

Descriptives

Code

region <fctr>	status_mean <dbl>
CN	23.52
UK	13.14

2 rows

Exploratory Analyses

Creativity

UK participants

Code

```
## Anova Table (Type 3 tests)
##
## Response: creat
##     Effect     df   MSE         F   pes p.value
## 1      sex 1, 235 99.38      0.12 .0005     .73
## 2     term 1, 235 30.50 42.55 ***   .15  <.0001
## 3 sex:term 1, 235 30.50      0.03 .0001     .85
## ---
## Signif. codes:  0 '***' 0.001 '**' 0.01 '*' 0.05 '+' 0.1 ' ' 1
```

CN participants

Code

```
## Anova Table (Type 3 tests)
##
## Response: creat
##      Effect     df   MSE         F   pes p.value
## 1       sex 1, 289 81.52    5.08 *     .02     .02
## 2      term 1, 289 35.67 34.73 ***     .11  <.0001
## 3 sex:term 1, 289 35.67      0.00 <.0001     .97
## ---
## Signif. codes:  0 '***' 0.001 '**' 0.01 '*' 0.05 '+' 0.1 ' ' 1
```

Descriptives

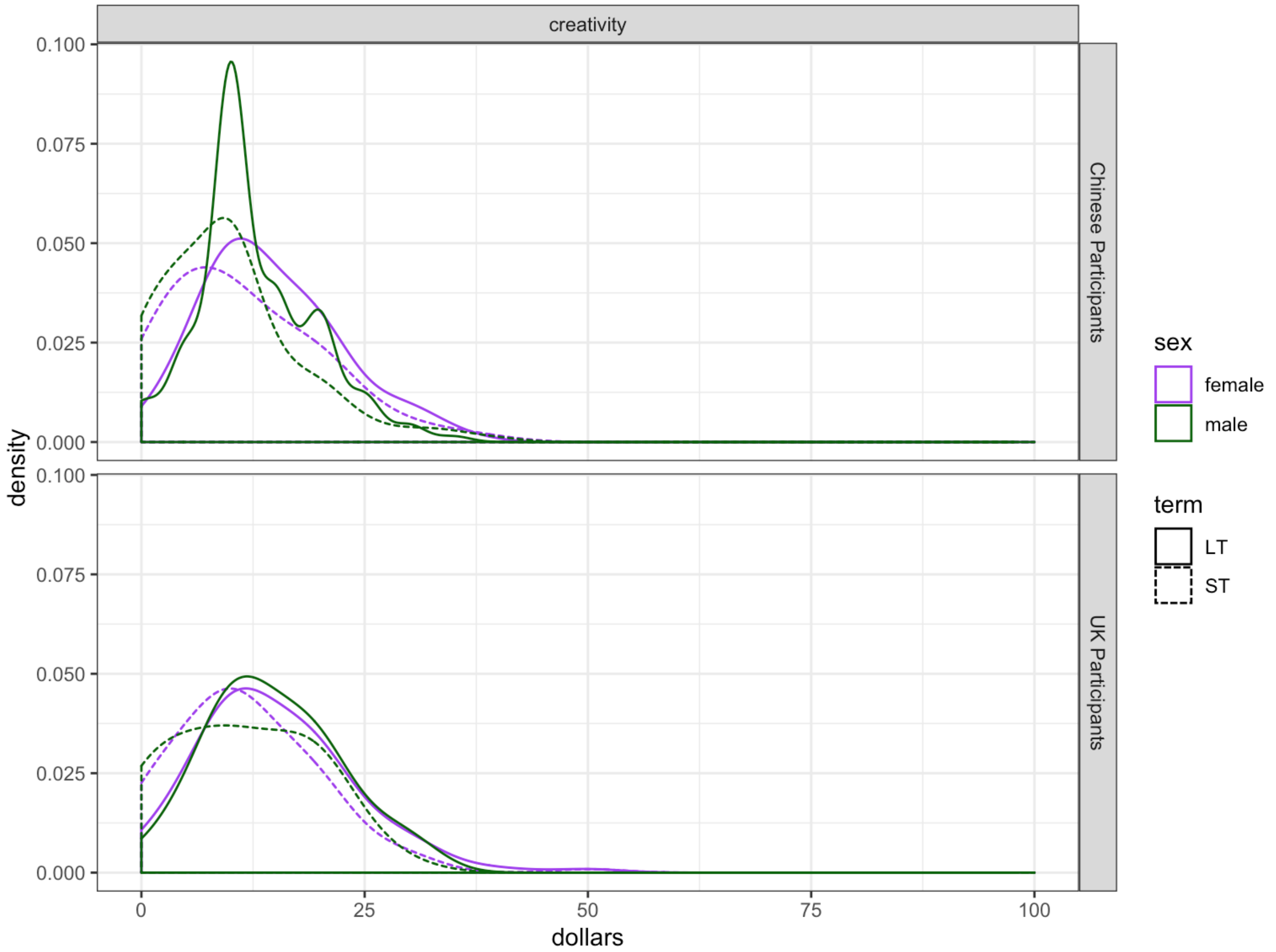

Code

sex <fctr>	region <fctr>	LT <dbl>	ST <dbl>	creat_term_dif <dbl>
female	CN	14.44	11.50	2.94
female	UK	14.97	11.75	3.22
male	CN	12.73	9.83	2.90
male	UK	14.74	11.34	3.40

4 rows

Kindness

UK participants

Code

```
## Anova Table (Type 3 tests)
##
## Response: kind
##     Effect     df    MSE         F pes p.value
## 1      sex 1, 229 174.95 19.87 *** .08  <.0001
## 2     term 1, 229  65.90 90.63 *** .28  <.0001
## 3 sex:term 1, 229  65.90    4.36 * .02     .04
## ---
## Signif. codes:  0 '***' 0.001 '**' 0.01 '*' 0.05 '+' 0.1 ' ' 1
```

CN participants

Code

```
## Anova Table (Type 3 tests)
##
## Response: kind
##     Effect     df    MSE         F   pes p.value
## 1      sex 1, 307 182.21      1.95  .006     .16
## 2     term 1, 307  74.39 88.48 ***   .22  <.0001
## 3 sex:term 1, 307  74.39      0.04 .0001     .84
## ---
## Signif. codes:  0 '***' 0.001 '**' 0.01 '*' 0.05 '+' 0.1 ' ' 1
```

Descriptives

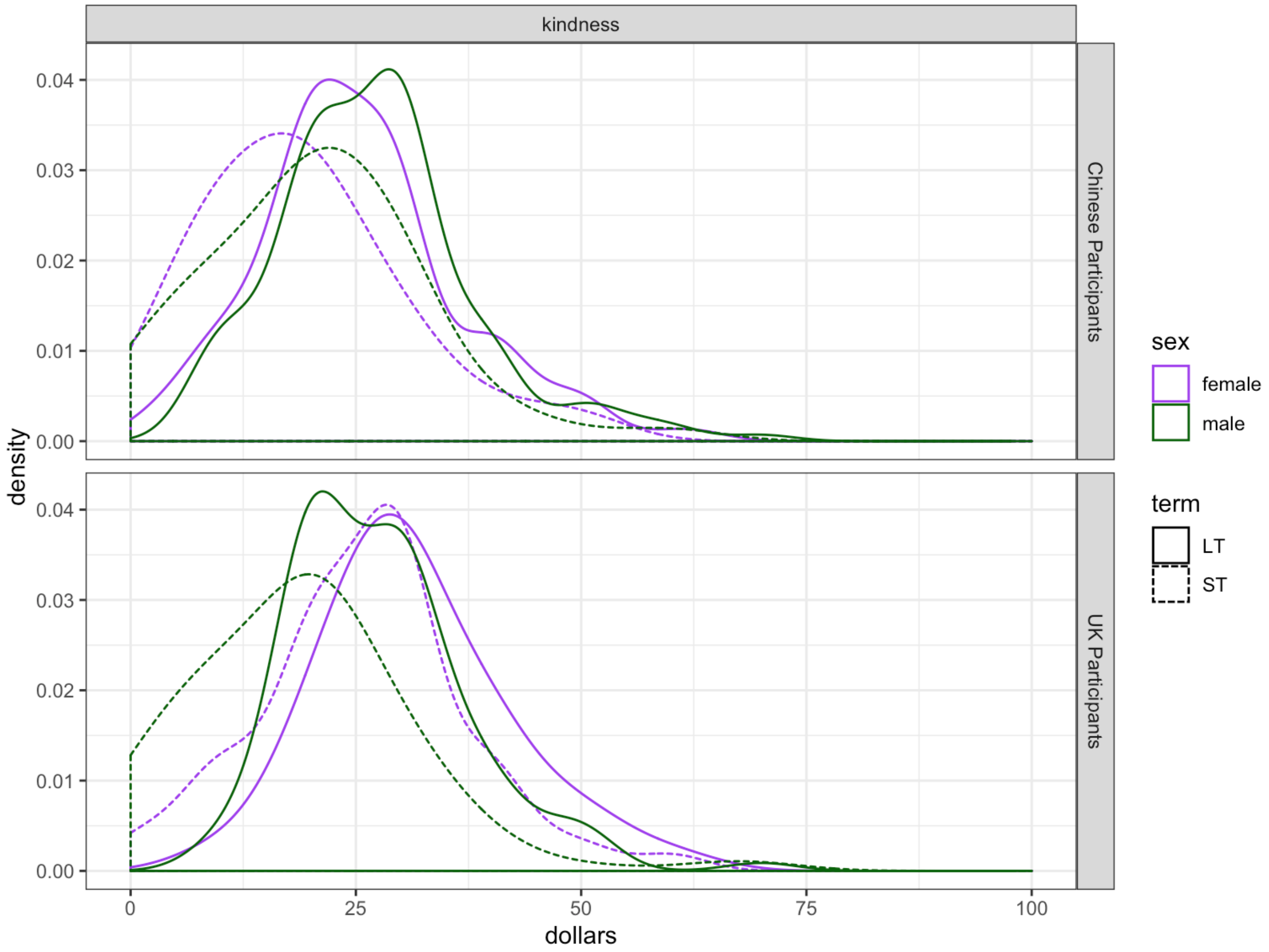

Code

region	term	male	female	kind_sex_dif
<fctr>	<fctr>	<dbl>	<dbl>	<dbl>
CN	LT	27.03	25.37	1.66
CN	ST	20.35	18.98	1.37
UK	LT	27.55	31.46	-3.91
UK	ST	18.78	25.84	-7.06

4 rows

Liveliness

UK participants

Code

```
## Anova Table (Type 3 tests)
##
## Response: lively
##     Effect     df   MSE       F   pes p.value
## 1      sex 1, 175 84.61 8.62 **   .05    .004
## 2     term 1, 175 34.43    0.49  .003     .48
## 3 sex:term 1, 175 34.43    0.03 .0002     .86
## ---
## Signif. codes:  0 '***' 0.001 '**' 0.01 '*' 0.05 '+' 0.1 ' ' 1
```

CN participants

Code

```
## Anova Table (Type 3 tests)
##
## Response: lively
##     Effect     df    MSE         F  pes p.value
## 1      sex 1, 292 100.73 14.27 ***  .05   .0002
## 2     term 1, 292  32.13      1.42 .005     .23
## 3 sex:term 1, 292  32.13    3.79 +  .01     .05
## ---
## Signif. codes:  0 '***' 0.001 '**' 0.01 '*' 0.05 '+' 0.1 ' ' 1
```

Descriptives

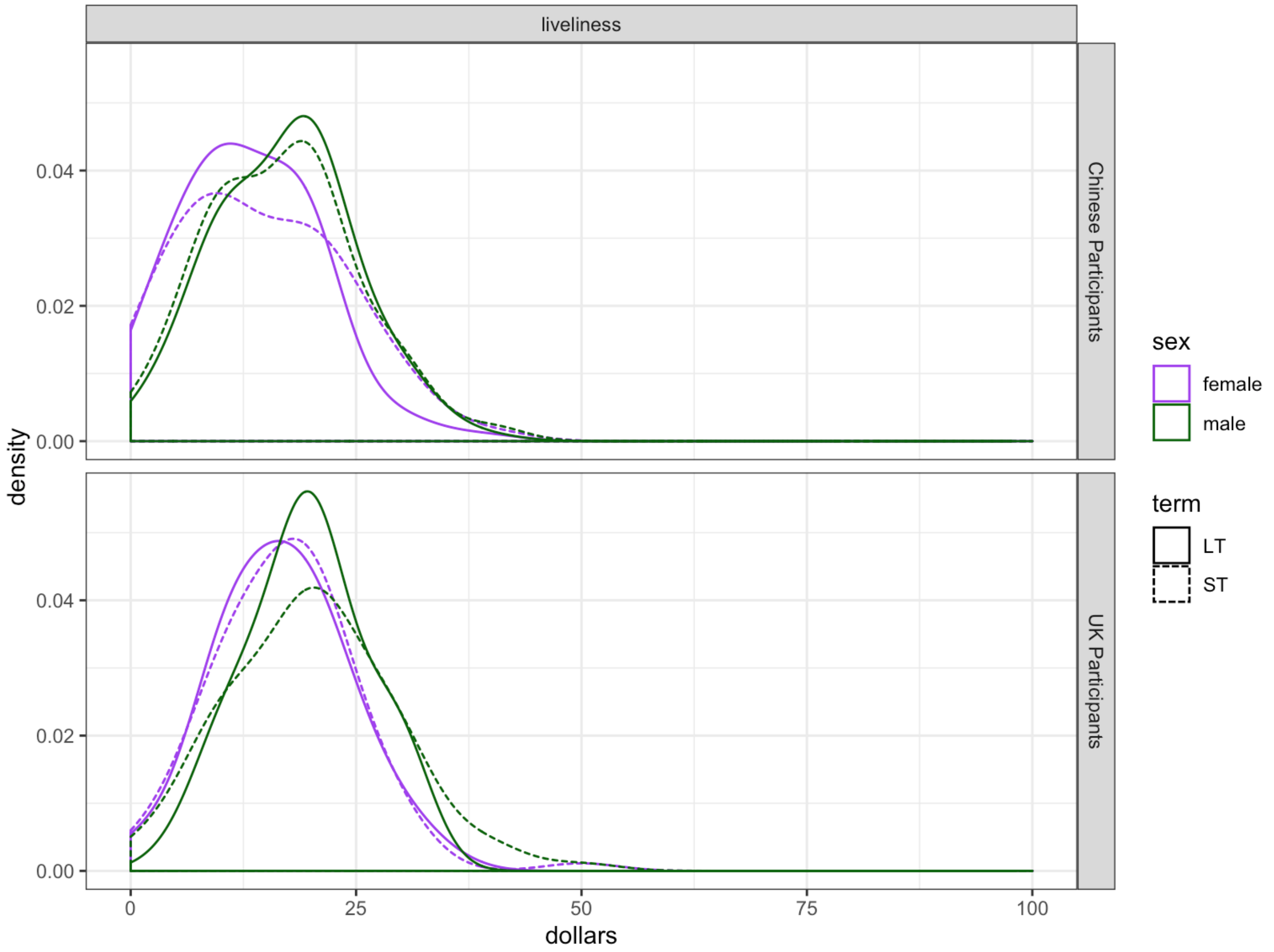

Code

region <fctr>	term <fctr>	male <dbl>	female <dbl>	lively_sex_dif <dbl>
CN	LT	17.12	13.08	4.04
CN	ST	16.76	14.55	2.22
UK	LT	19.53	16.77	2.76
UK	ST	20.08	17.09	2.98

4 rows

Age and Ideal Partner Age

Code

UK Participants

Code

```
## Anova Table (Type 3 tests)
##
## Response: age_diff
##     Effect     df  MSE          F   pes p.value
## 1      sex 1, 245 4.17 104.99 ***   .30  <.0001
## 2     term 1, 245 2.21  27.86 ***   .10  <.0001
## 3 sex:term 1, 245 2.21       0.20 .0008     .66
## ---
## Signif. codes:  0 '***' 0.001 '**' 0.01 '*' 0.05 '+' 0.1 ' ' 1
```

CN Participants

Code

```
## Anova Table (Type 3 tests)
##
## Response: age_diff
##     Effect     df   MSE          F pes p.value
## 1      sex 1, 310 11.89  83.94 *** .21  <.0001
## 2     term 1, 310  5.08 165.14 *** .35  <.0001
## 3 sex:term 1, 310  5.08     5.09 * .02     .02
## ---
## Signif. codes:  0 '***' 0.001 '**' 0.01 '*' 0.05 '+' 0.1 ' ' 1
```

Descriptives

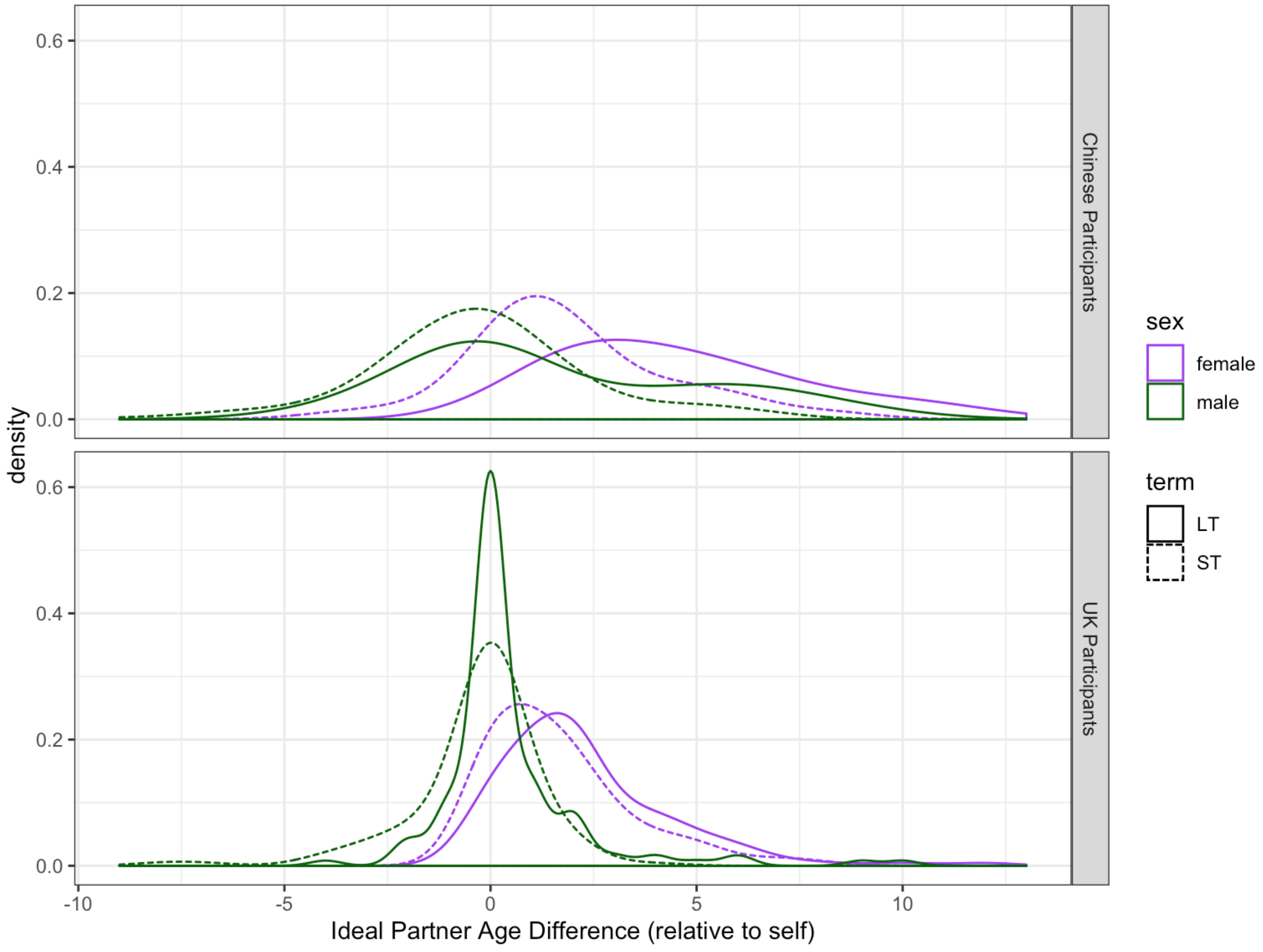
density
Ideal Partner Age Difference (relative to self)
Chinese Participants
UK Participants
sex
female
male
term
LT
ST
0.6
0.4
0.2
0.0
-10
-5
0
5
10

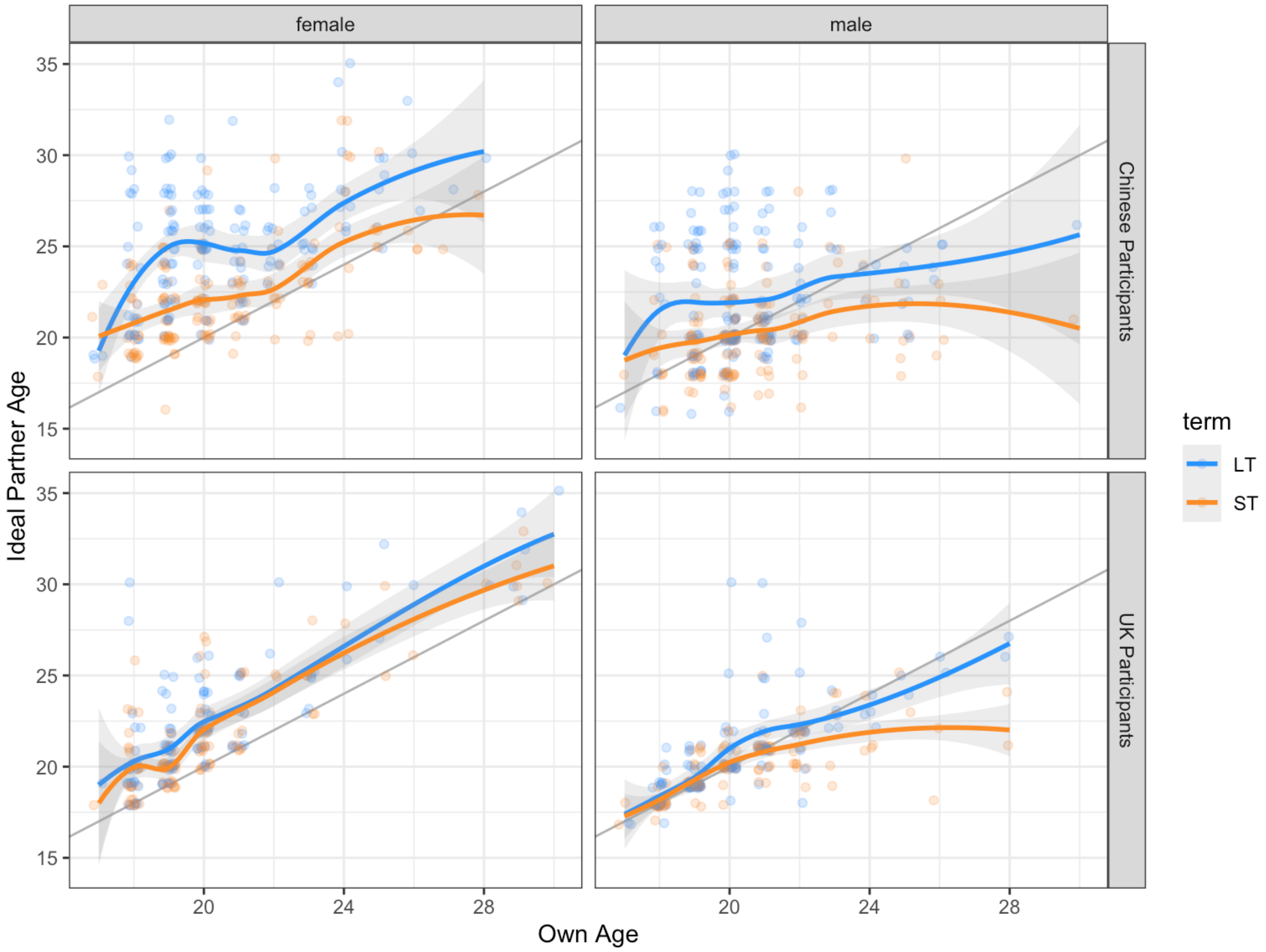
female
male
Chinese Participants
UK Participants
Ideal Partner Age
Own Age
term
LT
ST
35
30
25
20
15
20
24
28

Code

region <fctr>	term <fctr>	male <dbl>	female <dbl>	age_diff_sex_dif <dbl>
CN	LT	1.63	4.57	-2.94
CN	ST	-0.28	1.84	-2.13
UK	LT	0.46	2.29	-1.82
UK	ST	-0.30	1.64	-1.94

4 rows

Code

sex <fctr>	region <fctr>	LT <dbl>	ST <dbl>	age_diff_term_dif <dbl>
female	CN	4.57	1.84	2.73
female	UK	2.29	1.64	0.65
male	CN	1.63	-0.28	1.91
male	UK	0.46	-0.30	0.77

4 rows

Comparision among traits

Female mate preference for each trait in short-term relationships

Code

```
##               Df Sum Sq Mean Sq F value Pr(>F)    
## traits         4  66416   16604   131.1 <2e-16 ***
## Residuals   1221 154699     127                   
## ---
## Signif. codes:  0 '***' 0.001 '**' 0.01 '*' 0.05 '.' 0.1 ' ' 1
## 139 observations deleted due to missingness
```

Code

```
##   Tukey multiple comparisons of means
##     95% family-wise confidence level
##
## Fit: aov(formula = dollars ~ traits, data = .)
##
## $traits
##                     diff        lwr        upr     p adj
## creat-att     -21.815051 -24.650591 -18.979510 0.0000000
## kind-att      -11.486042 -14.296608  -8.675476 0.0000000
## lively-att    -17.918211 -20.830782 -15.005639 0.0000000
## status-att    -17.284186 -20.099611 -14.468760 0.0000000
## kind-creat     10.329009   7.626356  13.031661 0.0000000
## lively-creat    3.896840   1.088259   6.705421 0.0014809
## status-creat    4.530865   1.823159   7.238571 0.0000524
## lively-kind    -6.432169  -9.215533  -3.648804 0.0000000
## status-kind    -5.798144  -8.479685  -3.116603 0.0000000
## status-lively   0.634025  -2.154246   3.422296 0.9717202
```

Female mate preference for each trait in long-term relationships

Code

```
##               Df Sum Sq Mean Sq F value Pr(>F)
## traits         4  31823    7956   80.22 <2e-16 ***
## Residuals   1221 121095      99
## ---
## Signif. codes:  0 '***' 0.001 '**' 0.01 '*' 0.05 '.' 0.1 ' ' 1
## 139 observations deleted due to missingness
```

Code

```
##   Tukey multiple comparisons of means
##     95% family-wise confidence level
##
## Fit: aov(formula = dollars ~ traits, data = .)
##
## $traits
##                      diff        lwr        upr     p adj
## creat-att      -6.3143314  -8.823064  -3.805599 0.0000000
## kind-att        7.0007783   4.514142   9.487415 0.0000000
## lively-att     -6.5196629  -9.096549  -3.942777 0.0000000
## status-att      0.5503677  -1.940568   3.041304 0.9745427
## kind-creat     13.3151098  10.923949  15.706271 0.0000000
## lively-creat   -0.2053314  -2.690212   2.279549 0.9994246
## status-creat    6.8646992   4.469067   9.260331 0.0000000
## lively-kind   -13.5204412 -15.983011 -11.057871 0.0000000
## status-kind    -6.4504106  -8.822893  -4.077928 0.0000000
## status-lively   7.0700306   4.603119   9.536942 0.0000000
```

Male mate preference for each trait in short-term relationships

Code

```
##                Df Sum Sq Mean Sq F value Pr(>F)    
## traits          4 164692   41173   272.2 <2e-16 ***
## Residuals    1315 198921     151                   
## ---
## Signif. codes:  0 '***' 0.001 '**' 0.01 '*' 0.05 '.' 0.1 ' ' 1
## 175 observations deleted due to missingness
```

Code

```
##   Tukey multiple comparisons of means
##     95% family-wise confidence level
## 
## Fit: aov(formula = dollars ~ traits, data = .)
## 
## $traits
##                      diff        lwr        upr     p adj
## creat-att     -30.8644339 -33.787720 -27.941148 0.0000000
## kind-att      -21.6760017 -24.594177 -18.757826 0.0000000
## lively-att    -23.3607843 -26.369451 -20.352118 0.0000000
## status-att    -30.4973157 -33.428391 -27.566241 0.0000000
## kind-creat      9.1884322   6.323329  12.053536 0.0000000
## lively-creat    7.5036496   4.546431  10.460869 0.0000000
## status-creat    0.3671183  -2.511122   3.245359 0.9968403
## lively-kind    -1.6847826  -4.636950   1.267385 0.5242252
## status-kind    -8.8213140 -11.694365  -5.948263 0.0000000
## status-lively  -7.1365314 -10.101450  -4.171612 0.0000000
```

Male mate preference for each trait in long-term relationships

Code

```
##                Df Sum Sq Mean Sq F value Pr(>F)    
## traits          4  50425   12606   129.9 <2e-16 ***
## Residuals    1315 127640      97                   
## ---
## Signif. codes:  0 '***' 0.001 '**' 0.01 '*' 0.05 '.' 0.1 ' ' 1
## 175 observations deleted due to missingness
```

Code

```
##   Tukey multiple comparisons of means
##     95% family-wise confidence level
##
## Fit: aov(formula = dollars ~ traits, data = .)
##
## $traits
##                      diff         lwr         upr     p adj
## creat-att     -14.5756405 -16.9172999 -12.2339810 0.0000000
## kind-att       -0.9497016  -3.2872677   1.3878645 0.8014554
## lively-att    -10.1796850 -12.5897377  -7.7696323 0.0000000
## status-att    -13.2329788 -15.5808778 -10.8850798 0.0000000
## kind-creat     13.6259389  11.3308855  15.9209922 0.0000000
## lively-creat    4.3959555   2.0271143   6.7647967 0.0000045
## status-creat    1.3426617  -0.9629151   3.6482385 0.5035796
## lively-kind    -9.2299834 -11.5947783  -6.8651884 0.0000000
## status-kind   -12.2832772 -14.5846964  -9.9818579 0.0000000
## status-lively  -3.0532938  -5.4283032  -0.6782845 0.0041968
```

Text

Attractiveness (Prediction 1)

Code

After data exclusions, 120 Chinese women, 142 Chinese men, 99 UK women, and 113 UK men could be included in the final analyses of physical attractiveness.

In both samples, the main effects of participant sex (Chinese: $F(1, 260) = 20.28, p < .001, \hat{\eta}_p^2 = .072$; UK: $F(1, 210) = 21.09, p < .001, \hat{\eta}_p^2 = .091$) and relationship context (Chinese: $F(1, 260) = 151.54, p < .001, \hat{\eta}_p^2 = .368$; UK: $F(1, 210) = 137.80, p < .001, \hat{\eta}_p^2 = .396$) were significant. Men allocated more mate dollars to physical attractiveness than women did and people allocated more mate dollars to physical attractiveness for short-term relationships than they did for long-term relationships (see Table 1). The interaction was not significant in either sample (Chinese: $F(1, 260) = 0.18, p = .671, \hat{\eta}_p^2 = .001$; UK: $F(1, 210) = 1.73, p = .189, \hat{\eta}_p^2 = .008$). These data support Prediction 1a, but not Prediction 1b.

Social Status (Prediction 2)

Code

After data exclusions, 144 Chinese women, 151 Chinese men, 118 UK women, and 120 UK men could be included in the final analyses of social status.

In both samples, the main effects of participant sex (Chinese: $F(1, 293) = 68.63, p < .001, \hat{\eta}_p^2 = .190$; UK: $F(1, 236) = 12.01, p = .001, \hat{\eta}_p^2 = .048$) and relationship context (Chinese: $F(1, 293) = 74.98, p < .001, \hat{\eta}_p^2 = .204$; UK: $F(1, 236) = 31.11, p < .001, \hat{\eta}_p^2 = .116$) were significant. Women allocated more mate dollars to social status than men did and people allocated more mate dollars to social status for long-term relationships than they did for short-term relationships

(see Table 1). The interaction was not significant in either sample (Chinese: $F(1, 293) = 3.10$, $p = .080$, $\hat{\eta}_p^2 = .010$; UK: $F(1, 236) = 0.01$, $p = .923$, $\hat{\eta}_p^2 = .000$). These data support Prediction 2a, but not Prediction 2b.

Social Status (Prediction 3)

Code

Consistent with Prediction 3, Chinese women allocated significantly more mate dollars to social status for long-term relationships than did UK women ($F(1, 260) = 93.52$, $p < .001$, $\hat{\eta}_p^2 = .265$).

Creativity

Code

Analyses of creativity (Table 3) showed significant effects of relationship context in both samples (Chinese: $F(1, 289) = 34.73$, $p < .001$, $\hat{\eta}_p^2 = .107$; UK: $F(1, 235) = 42.55$, $p < .001$, $\hat{\eta}_p^2 = .153$) and a significant effect of participant sex in the Chinese sample ($F(1, 289) = 5.08$, $p = .025$, $\hat{\eta}_p^2 = .017$), but not the UK sample ($F(1, 235) = 0.12$, $p = .730$, $\hat{\eta}_p^2 = .001$). The interaction was not significant in either sample (Chinese: $F(1, 289) = 0.00$, $p = .968$, $\hat{\eta}_p^2 = .000$; UK: $F(1, 235) = 0.03$, $p = .852$, $\hat{\eta}_p^2 = .000$).

Kindness

Code

Analyses of kindness (Table 4) showed significant effects of relationship context in both samples (Chinese: $F(1, 307) = 88.48$, $p < .001$, $\hat{\eta}_p^2 = .224$; UK: $F(1, 229) = 90.63$, $p < .001$, $\hat{\eta}_p^2 = .284$) and a significant effect of participant sex in the UK sample ($F(1, 229) = 19.87$, $p < .001$, $\hat{\eta}_p^2 = .080$), but not the Chinese sample ($F(1, 307) = 1.95$, $p = .164$, $\hat{\eta}_p^2 = .006$). The interaction was significant in the UK sample ($F(1, 229) = 4.36$, $p = .038$, $\hat{\eta}_p^2 = .019$), but not the Chinese sample ($F(1, 307) = 0.04$, $p = .837$, $\hat{\eta}_p^2 = .000$).

Liveliness

Code

Analyses of liveliness (Table 5) showed significant effects of participant sex in both samples (Chinese: $F(1, 292) = 14.27$, $p < .001$, $\hat{\eta}_p^2 = .047$; UK: $F(1, 175) = 8.62$, $p = .004$, $\hat{\eta}_p^2 = .047$) and no significant effect of relationship context in either sample (Chinese: $F(1, 292) = 1.42$, $p = .234$, $\hat{\eta}_p^2 = .005$; UK: $F(1, 175) = 0.49$, $p = .484$, $\hat{\eta}_p^2 = .003$). The interaction was not significant in either sample (Chinese: $F(1, 292) = 3.79$, $p = .053$, $\hat{\eta}_p^2 = .013$; UK: $F(1, 175) = 0.03$, $p = .858$, $\hat{\eta}_p^2 = .000$).

Age

Code

In both samples, the main effects of participant sex (Chinese: $F(1, 310) = 83.94, p < .001,$ $\hat{\eta}_p^2 = .213$; UK: $F(1, 245) = 104.99, p < .001, \hat{\eta}_p^2 = .300$) and relationship context (Chinese: $F(1, 310) = 165.14, p < .001, \hat{\eta}_p^2 = .348$; UK: $F(1, 245) = 27.86, p < .001, \hat{\eta}_p^2 = .102$) were significant. The interaction was significant in the Chinese sample ($F(1, 310) = 5.09, p = .025,$ $\hat{\eta}_p^2 = .016$), but not the UK sample ($F(1, 245) = 0.20, p = .658, \hat{\eta}_p^2 = .001$). These results are summarized in Table 6.

````
---
title: Chinese and UK Participants' Preferences for Physical Attractiveness
and Social Status in Potential Mates
author: "Zhang et al."
output:
  html_document:
    df_print: paged
    toc: yes
    code_folding: hide
---

```{r, message=FALSE}
knitr::opts_chunk$set(echo = TRUE, warning = FALSE, message = FALSE)
library(tidyverse) # for data wrangling
library(afex) # for SPSS-style ANOVA
#devtools::install_github("crsh/papaja")
library(papaja) # for APA-formatting of ANOVA results
```


## Data Processing

### Load Data

```{r, message = FALSE}
uk_demog <- read_csv("data/UK_demog.csv")
uk_st    <- read_csv("data/UK_short_term_dollars.csv")
uk_lt    <- read_csv("data/UK_long_term_dollars.csv")
uk_ideal <- read_csv("data/UK_ideal_partner_age.csv")
uk_word  <- read_csv("data/UK_translation_check.csv")

cn_demog <- read_csv("data/CN_demog.csv")
cn_st    <- read_csv("data/CN_short_term_dollars.csv")
cn_lt    <- read_csv("data/CN_long_term_dollars.csv")
cn_ideal <- read_csv("data/CN_ideal_partner_age.csv")
cn_word  <- read_csv("data/CN_translation_check.csv")
```

### Translation Checks

```{r translation-uk}
uk_tcheck <- uk_word %>%
  mutate(check_att = (`good looks` == 0) + (beauty == 0) + (hotness == 0),
         check_status = (rich == 1) + (wealthy == 1) + (`well-off` == 1),
         check_creat = (imaginative == 2) + (artisitic == 2) + (innovative
== 2),
         check_kind = (niceness == 3) + (selflessness == 3 ) + (giving ==
3),
         check_lively = (energetic == 4) + (outgoing == 4) + (sociable ==
4)
````
````

) %>%
  select(user_id, check_att:check_lively)
```

```{r translation-cn}
cn_tcheck <- cn_word %>%
  mutate(check_att = (`facial appearance` == 0) + (appearance == 0) + (charmness == 0),
         check_status = (income == 1) + (position == 1) + (power == 1),
         check_creat = (innovation == 2) + (intelligence == 2) + (reform == 2),
         check_kind = (caring == 3) + (compassion == 3 ) + (merciful == 3),
         check_lively = (enthusiastic == 4) + (outgoing == 4) + (cheerful == 4)
  ) %>%
  select(user_id, check_att:check_lively)
```

Join Data

```{r}
data_uk <- uk_demog %>%
  inner_join(uk_st, by = "user_id") %>%
  inner_join(uk_lt, by = "user_id") %>%
  inner_join(uk_ideal, by = "user_id") %>%
  inner_join(uk_tcheck, by = "user_id")

data_cn <- cn_demog %>%
  inner_join(cn_st, by = "user_id") %>%
  inner_join(cn_lt, by = "user_id") %>%
  inner_join(cn_ideal, by = "user_id") %>%
  inner_join(cn_tcheck, by = "user_id")

data.wide <- bind_rows(data_uk, data_cn)

# Only heterosexual participants
data.het <- data.wide %>%
  filter((sex == "male" & orientation == "women") |
         (sex == "female" & orientation == "men"))

#count(data.het, sex, region)

data.raw <- data.het %>%
  gather(trait, dollars, st_att:lt_partner_age) %>%
  separate(trait, c("term", "trait"), extra = "merge") %>%
  spread(trait, dollars) %>%
  mutate(term = toupper(term),
    age_diff = partner_age - age,
    sex = as.factor(sex),
    region = as.factor(region),

```
    term = as.factor(term)
)

```

### Data Exclusions

Ns for Raw Data (pre-exclusions)
```{r}

data.long <- data.raw %>%
  # put into long format for processing
  gather(var, val, check_att:check_lively, att:lively, status) %>%
  separate(var, c("var1", "trait"), fill = "left") %>%
  mutate(var1 = ifelse(is.na(var1), "dollars", var1)) %>%
  spread(var1, val)

pre.excl <- data.long %>%
  group_by(user_id, sex, region, trait) %>%
  summarise() %>%
  ungroup() %>%
  count(sex, region, trait) %>%
  spread(trait, n)

pre.excl
```

Keep only people who:

1. passed the definition check for the relevant word
2. <= 3 SDs from the mean for that sex
3. completed both LT and ST

Excluded due to failing definition check
```{r}
data.excl.check <- data.long %>%
  filter(check == 3)

excl.def <- anti_join(data.long, data.excl.check) %>%
  group_by(user_id, sex, region, trait) %>%
  summarise() %>%
  ungroup() %>%
  count(sex, region, trait) %>%
  spread(trait, n, fill = 0)

excl.def
```

Excluded due to not responding to dollars question

````
```{r}
# only keep if responded to dollars Q for both LT & ST for a trait
data.excl.missingdollars <- data.excl.check %>%
  filter(!is.na(dollars)) %>%
  group_by(user_id, trait) %>%
  filter(n() == 2) %>% # must have both LT and ST after this
  ungroup()

excl.missing <- anti_join(data.excl.check, data.excl.missingdollars) %>%
  group_by(user_id, sex, region, trait) %>%
  summarise() %>%
  ungroup() %>%
  count(sex, region, trait) %>%
  spread(trait, n, fill = 0)

excl.missing
```
````

Excluded due to not completing both LT and ST after outlier exclusion

````
```{r}
  # only keep if <= 3SD from the sex-specific mean
data.excl <- data.excl.missingdollars %>%
  group_by(sex, trait) %>%
  filter(
    dollars >= mean(dollars) - 3*sd(dollars),
    dollars <= mean(dollars) + 3*sd(dollars)
  ) %>%
  ungroup() %>%
  group_by(user_id, trait) %>%
  filter(n() == 2) %>% # must have both LT and ST
  ungroup()

excl.outlier <- anti_join(data.excl.missingdollars, data.excl) %>%
  group_by(user_id, sex, region, trait) %>%
  summarise() %>%
  ungroup() %>%
  count(sex, region, trait) %>%
  spread(trait, n, fill = 0)

excl.outlier
```
````

````
```{r}
# put back in wide format
data <- data.excl %>%
````
````

```
  select(-check) %>%
  spread(trait, dollars)

```

General Descriptives

Number of post-exclusion participants per region, sex and trait:
```{r}
post.excl <- data.excl %>%
  group_by(user_id, sex, region, trait) %>%
  summarise() %>%
  ungroup() %>%
  count(sex, region, trait) %>%
  spread(trait, n, fill = 0)

post.excl
```



```{r}
excl <- mutate(pre.excl, excl = "pre") %>%
  bind_rows(mutate(excl.def, excl = "def")) %>%
  bind_rows(mutate(excl.missing, excl = "missing")) %>%
  bind_rows(mutate(excl.outlier, excl = "outlier")) %>%
  bind_rows(mutate(post.excl, excl = "post")) %>%
  gather(trait, val, att:status) %>%
  spread(excl, val, fill = 0) %>%
  mutate(total_excluded = def+missing+outlier,
         calc_post = pre-total_excluded) %>%
  select(sex:trait, pre, def, missing, outlier, total_excluded, post) %>%
  arrange(trait, sex, region)

excl
```

Means and SDs per per region, sex and trait (post-exclusions):
```{r}
data %>%
  select(sex, region, term, att:lively, status) %>%
  gather(trait, value, att:status) %>%
  group_by(sex, region, term, trait) %>%
  summarise(n = n(),
            mean = mean(value, na.rm = TRUE) %>%
              round(2) %>% as.character() %>%
              str_pad(5, "right", 0),
            sd = sd(value, na.rm = TRUE) %>% round(2)) %>%
  mutate(mean_sd = paste0(mean, " (", sd, ")")) %>%
  select(-mean, -sd) %>%
  spread(trait, mean_sd) %>%
```

````
  rename(attractiveness = att,
         creativity = creat,
         kindness = kind,
         liveliness = lively)
```

### Visualisations

```{r, fig.width = 8, fig.height = 5}

data %>%
  gather(trait, dollars, att, status) %>%
  mutate(trait = recode(trait, "att" = "attractiveness",
                        "creat" = "creativity",
                        "kind" = "kindness",
                        "lively" = "liveliness",
                        "status" = "social status"),
         region = recode(region,
                         "CN" = "Chinese Participants",
                         "UK" = "UK Participants")
  ) %>%
  ggplot(aes(dollars, color = sex, linetype = term)) +
  geom_density(adjust = 1.5) +
  facet_grid(region ~ trait) +
  xlim(0,100) +
  theme_bw() +
  scale_color_manual(values = c("purple", "darkgreen"))

ggsave("distributions.png", width = 8, height = 5)

```



```{r, fig.width = 12, fig.height = 5}

data %>%
  gather(trait, dollars, creat, kind, lively,
         factor_key = TRUE) %>%
  mutate(trait = recode(trait, "att" = "attractiveness",
                        "creat" = "creativity",
                        "kind" = "kindness",
                        "lively" = "liveliness",
                        "status" = "social status"),
         region = recode(region,
                         "CN" = "Chinese Participants",
                         "UK" = "UK Participants")
  ) %>%
  ggplot(aes(dollars, color = sex, linetype = term)) +
  geom_density(adjust = 1.5) +
  facet_grid(region ~ trait) +
````

```
  xlim(0,100) +
  theme_bw() +
  scale_color_manual(values = c("purple", "darkgreen"))

ggsave("distributions_ckl.png", width = 12, height = 5)

```

Confirmatory Analyses

Prediction 1 (Attractiveness)

1A. Men will allocate more mate dollars to physical attractiveness than women in both Chinese and UK samples.

1B. This sex difference will be particularly pronounced when choosing for potential short-term partners than for potential long-term partners.

UK Participants
```{r}
pred1.UK <- aov_4(att ~ sex * term + (1 + term | user_id),
                  data = filter(data, region == "UK"),
                  anova_table = list(es = "pes"))

pred1.UK
```

CN Participants
```{r}
pred1.CN <- aov_4(att ~ sex * term + (1 + term | user_id),
                  data = filter(data, region == "CN"),
                  anova_table = list(es = "pes"))

pred1.CN
```

Descriptives

```{r, echo = FALSE, fig.width = 8, fig.height = 6}

data %>%
  gather(trait, dollars, att) %>%
  mutate(trait = recode(trait, "att" = "attractiveness",
                        "creat" = "creativity",
                        "kind" = "kindness",
                        "lively" = "liveliness",
                        "status" = "social status"),
         region = recode(region,
                         "CN" = "Chinese Participants",
```

```
                       "UK" = "UK Participants")
  ) %>%
  ggplot(aes(dollars, color = sex, linetype = term)) +
  geom_density(adjust = 1.5) +
  facet_grid(region ~ trait) +
  xlim(0,100) +
  theme_bw() +
  scale_color_manual(values = c("purple", "darkgreen"))
```

```{r}
data %>%
  group_by(sex, region) %>%
  summarise(att_mean = mean(att, na.rm = TRUE)) %>%
  spread(sex, att_mean) %>%
  mutate(att_sex_dif = male - female) %>%
  select(region, male, female, att_sex_dif) %>%
  mutate_if(is.numeric, round, 2)
```

```{r}
data %>%
  group_by(sex, region, term) %>%
  summarise(att_mean = mean(att, na.rm = TRUE)) %>%
  ungroup() %>%
  spread(sex, att_mean) %>%
  mutate(att_sex_dif = male - female) %>%
  select(region, term, male, female, att_sex_dif) %>%
  mutate_if(is.numeric, round, 2)
```

Prediction 2 (Status I)

2A. Women will allocate significantly more mate dollars to social status than men in both the UK and Chinese samples.

2B. This sex difference will be significantly more pronounced when choosing for potential long-term partners than short-term partners.

UK Participants

```{r}
pred2.UK <- aov_4(status ~ sex * term + (1 + term | user_id),
                  data = filter(data, region == "UK"),
                  anova_table = list(es = "pes"))

pred2.UK
```

````
### CN Participants
```{r}
pred2.CN <- aov_4(status ~ sex * term + (1 + term | user_id),
                  data = filter(data, region == "CN"),
                  anova_table = list(es = "pes"))

pred2.CN
```

### Descriptives

```{r, echo = FALSE, fig.width = 8, fig.height = 6}

data %>%
  gather(trait, dollars, status) %>%
  mutate(trait = recode(trait, "att" = "attractiveness",
                        "creat" = "creativity",
                        "kind" = "kindness",
                        "lively" = "liveliness",
                        "status" = "social status"),
         region = recode(region,
                         "CN" = "Chinese Participants",
                         "UK" = "UK Participants")
  ) %>%
  ggplot(aes(dollars, color = sex, linetype = term)) +
  geom_density(adjust = 1.5) +
  facet_grid(region ~ trait) +
  xlim(0,100) +
  theme_bw() +
  scale_color_manual(values = c("purple", "darkgreen"))
```

```{r}
data %>%
  group_by(sex, region) %>%
  summarise(status_mean = mean(status, na.rm = TRUE)) %>%
  spread(sex, status_mean) %>%
  mutate(status_sex_dif = male - female) %>%
  select(region, male, female, status_sex_dif) %>%
  mutate_if(is.numeric, round, 2)
```


```{r}
data %>%
  group_by(sex, region, term) %>%
  summarise(status_mean = mean(status, na.rm = TRUE)) %>%
  ungroup() %>%
  spread(sex, status_mean) %>%
  mutate(status_sex_dif = male - female) %>%
````
````

```
  select(region, term, male, female, status_sex_dif) %>%
  mutate_if(is.numeric, round, 2)
```

Prediction 3 (Status II)

Chinese women will allocate significantly more mate dollars to social status than UK women will in the long-term context.

```{r}
pred3 <- aov_4(status ~ region + (1 | user_id),
                  data = filter(data, sex == "female", term == "LT"),
                  anova_table = list(es = "pes"))

pred3
```

Descriptives
```{r}
data %>%
  filter(sex == "female") %>%
  group_by(region) %>%
  summarise(status_mean = mean(status, na.rm = TRUE)) %>%
  mutate_if(is.numeric, round, 2)

```

Exploratory Analyses

Creativity

UK participants
```{r}

creat.UK <- aov_4(creat ~ sex * term + (1 + term | user_id),
                  data = filter(data, region == "UK"),
                  anova_table = list(es = "pes"))

creat.UK

```

CN participants
```{r}

creat.CN <- aov_4(creat ~ sex * term + (1 + term | user_id),
                  data = filter(data, region == "CN"),
                  anova_table = list(es = "pes"))
```

````
creat.CN
```

### Descriptives

```{r, echo = FALSE, fig.width = 8, fig.height = 6}

data %>%
  gather(trait, dollars, creat) %>%
  mutate(trait = recode(trait, "att" = "attractiveness",
                       "creat" = "creativity",
                       "kind" = "kindness",
                       "lively" = "liveliness",
                       "status" = "social status"),
         region = recode(region,
                        "CN" = "Chinese Participants",
                        "UK" = "UK Participants")
  ) %>%
  ggplot(aes(dollars, color = sex, linetype = term)) +
  geom_density(adjust = 1.5) +
  facet_grid(region ~ trait) +
  xlim(0,100) +
  theme_bw() +
  scale_color_manual(values = c("purple", "darkgreen"))
```

```{r}
data %>%
  group_by(sex, term, region) %>%
  summarise(creat_mean = mean(creat, na.rm = TRUE)) %>%
  ungroup() %>%
  spread(term, creat_mean) %>%
  mutate(creat_term_dif = LT - ST) %>%
  select(sex, region, LT, ST, creat_term_dif) %>%
  mutate_if(is.numeric, round, 2)
```

## Kindness

### UK participants
```{r}

kind.UK <- aov_4(kind ~ sex * term + (1 + term | user_id),
                  data = filter(data, region == "UK"),
                  anova_table = list(es = "pes"))
kind.UK
```

### CN participants
```{r}
````

kind.CN <- aov_4(kind ~ sex * term + (1 + term | user_id),
                  data = filter(data, region == "CN"),
                  anova_table = list(es = "pes"))

kind.CN
```

### Descriptives

```{r, echo = FALSE, fig.width = 8, fig.height = 6}

data %>%
  gather(trait, dollars, kind) %>%
  mutate(trait = recode(trait, "att" = "attractiveness",
                        "creat" = "creativity",
                        "kind" = "kindness",
                        "lively" = "liveliness",
                        "status" = "social status"),
         region = recode(region,
                         "CN" = "Chinese Participants",
                         "UK" = "UK Participants")
  ) %>%
  ggplot(aes(dollars, color = sex, linetype = term)) +
  geom_density(adjust = 1.5) +
  facet_grid(region ~ trait) +
  xlim(0,100) +
  theme_bw() +
  scale_color_manual(values = c("purple", "darkgreen"))
```

```{r}
data %>%
  group_by(sex, region, term) %>%
  summarise(kind_mean = mean(kind, na.rm = TRUE)) %>%
  ungroup() %>%
  spread(sex, kind_mean) %>%
  mutate(kind_sex_dif = male - female) %>%
  select(region, term, male, female, kind_sex_dif) %>%
  mutate_if(is.numeric, round, 2)
```

Liveliness

UK participants
```{r}

lively.UK <- aov_4(lively ~ sex * term + (1 + term | user_id),
                  data = filter(data, region == "UK"),
                  anova_table = list(es = "pes"))

lively.UK
```

CN participants
```{r}

lively.CN <- aov_4(lively ~ sex * term + (1 + term | user_id),
                  data = filter(data, region == "CN"),
                  anova_table = list(es = "pes"))

lively.CN
```

Descriptives

```{r, echo = FALSE, fig.width = 8, fig.height = 6}

data %>%
  gather(trait, dollars, lively) %>%
  mutate(trait = recode(trait, "att" = "attractiveness",
                        "creat" = "creativity",
                        "kind" = "kindness",
                        "lively" = "liveliness",
                        "status" = "social status"),
         region = recode(region,
                         "CN" = "Chinese Participants",
                         "UK" = "UK Participants")
  ) %>%
  ggplot(aes(dollars, color = sex, linetype = term)) +
  geom_density(adjust = 1.5) +
  facet_grid(region ~ trait) +
  xlim(0,100) +
  theme_bw() +
  scale_color_manual(values = c("purple", "darkgreen"))
```

```{r}
data %>%
  group_by(sex, region, term) %>%
  summarise(lively_mean = mean(lively, na.rm = TRUE)) %>%
  ungroup() %>%
  spread(sex, lively_mean) %>%
  mutate(lively_sex_dif = male - female) %>%
  select(region, term, male, female, lively_sex_dif) %>%
  mutate_if(is.numeric, round, 2)
```

Age and Ideal Partner Age
```

````
```{r}
data.age <- data %>%
  filter(!is.na(age_diff)) %>%
  group_by(sex, term) %>%
  filter(
    age_diff >= mean(age_diff) - 3*sd(age_diff),
    age_diff <= mean(age_diff) + 3*sd(age_diff)
  ) %>%
  ungroup() %>%
  group_by(user_id) %>%
  filter(n() == 2) %>% # must have both LT and ST
  ungroup()
```


### UK Participants
```{r}
age.UK <- aov_4(age_diff ~ sex * term + (1 + term | user_id),
                data = filter(data.age, region == "UK"),
                anova_table = list(es = "pes"))
age.UK

```


### CN Participants
```{r}

age.CN <- aov_4(age_diff ~ sex * term + (1 + term | user_id),
                data = filter(data.age, region == "CN"),
                anova_table = list(es = "pes"))
age.CN
```

### Descriptives

```{r, echo = FALSE, fig.width = 8, fig.height = 6}

data.age %>%
  mutate(region = recode(region,
                         "CN" = "Chinese Participants",
                         "UK" = "UK Participants")
  ) %>%
  ggplot(aes(age_diff, color = sex, linetype = term)) +
  geom_density(adjust = 1.5) +
  facet_grid(region ~.) +
  xlab("Ideal Partner Age Difference (relative to self)") +
  theme_bw() +
  scale_color_manual(values = c("purple", "darkgreen"))
```
````

```{r, echo = FALSE, fig.width = 8, fig.height = 6}

data.age %>%
  mutate(region = recode(region,
                         "CN" = "Chinese Participants",
                         "UK" = "UK Participants")
  ) %>%
  ggplot(aes(age, partner_age, color = term)) +
  geom_abline(slope = 1, intercept = 0, color = "grey70") +
  geom_jitter(alpha = 0.2, height = 0.2, width = 0.2)+
  geom_smooth(alpha = 0.2)+
  facet_grid(region ~sex) +
  xlab("Own Age") +
  ylab("Ideal Partner Age") +
  theme_bw() +
  scale_color_manual(values = c("dodgerblue", "darkorange"))
```

```{r}
data.age %>%
  group_by(sex, region, term) %>%
  summarise(age_diff_mean = mean(age_diff, na.rm = TRUE)) %>%
  ungroup() %>%
  spread(sex, age_diff_mean) %>%
  mutate(age_diff_sex_dif = male - female) %>%
  select(region, term, male, female, age_diff_sex_dif) %>%
  mutate_if(is.numeric, round, 2)
```

```{r}
data.age %>%
  group_by(sex, term, region) %>%
  summarise(age_diff_mean = mean(age_diff, na.rm = TRUE)) %>%
  ungroup() %>%
  spread(term, age_diff_mean) %>%
  mutate(age_diff_term_dif = LT - ST) %>%
  select(sex, region, LT, ST, age_diff_term_dif) %>%
  mutate_if(is.numeric, round, 2)

```

Comparision among traits

Female mate preference for each trait in short-term relationships

```{r}
female_ST <- data %>%
  filter(sex == "female" & term == "ST") %>%
```

  gather(traits, dollars, att:status) %>%
  aov(dollars ~ traits, data = .)

summary(female_ST)
TukeyHSD(female_ST)
```

### Female mate preference for each trait in long-term relationships

```{r}
female_LT <- data %>%
  filter(sex == "female" & term == "LT") %>%
  gather(traits, dollars, att:status) %>%
  aov(dollars ~ traits, data = .)

summary(female_LT)
TukeyHSD(female_LT)
```

Male mate preference for each trait in short-term relationships

```{r}
male_ST <- data %>%
  filter(sex == "male" & term == "ST") %>%
  gather(traits, dollars, att:status) %>%
  aov(dollars ~ traits, data = .)

summary(male_ST)
TukeyHSD(male_ST)
```

Male mate preference for each trait in long-term relationships

```{r}
male_LT <- data %>%
  filter(sex == "male" & term == "LT") %>%
  gather(traits, dollars, att:status) %>%
  aov(dollars ~ traits, data = .)

summary(male_LT)
TukeyHSD(male_LT)
```


Text

Attractiveness (Prediction 1)

```{r}

```
CNF <- filter(post.excl, sex == "female", region == "CN")$att
CNM <- filter(post.excl, sex == "male", region == "CN")$att
UKF <- filter(post.excl, sex == "female", region == "UK")$att
UKM <- filter(post.excl, sex == "male", region == "UK")$att

stat1.UK <- apa_print(pred1.UK, mse = FALSE, es = "pes")$full_result
stat1.CN <- apa_print(pred1.CN, mse = FALSE, es = "pes")$full_result
```

After data exclusions, `r CNF` Chinese women, `r CNM` Chinese men, `r UKF` UK women, and `r UKM` UK men could be included in the final analyses of physical attractiveness.

In both samples, the main effects of participant sex (Chinese: `r stat1.CN $sex`; UK: `r stat1.UK$sex`) and relationship context (Chinese: `r stat1.CN $term`; UK: `r stat1.UK$term`) were significant. Men allocated more mate dollars to physical attractiveness than women did and people allocated more mate dollars to physical attractiveness for short-term relationships than they did for long-term relationships (see Table 1). The interaction was not significant in either sample (Chinese: `r stat1.CN$sex_term`; UK: `r stat1.UK$sex_term`). These data support Prediction 1a, but not Prediction 1b.

Social Status (Prediction 2)

```{r}
CNF <- filter(post.excl, sex == "female", region == "CN")$status
CNM <- filter(post.excl, sex == "male", region == "CN")$status
UKF <- filter(post.excl, sex == "female", region == "UK")$status
UKM <- filter(post.excl, sex == "male", region == "UK")$status

stat2.UK <- apa_print(pred2.UK, mse = FALSE, es = "pes")$full_result
stat2.CN <- apa_print(pred2.CN, mse = FALSE, es = "pes")$full_result
```

After data exclusions, `r CNF` Chinese women, `r CNM` Chinese men, `r UKF` UK women, and `r UKM` UK men could be included in the final analyses of social status.

In both samples, the main effects of participant sex (Chinese: `r stat2.CN $sex`; UK: `r stat2.UK$sex`) and relationship context (Chinese: `r stat2.CN $term`; UK: `r stat2.UK$term`) were significant. Women allocated more mate dollars to social status than men did and people allocated more mate dollars to social status for long-term relationships than they did for short-term relationships (see Table 1). The interaction was not significant in either sample (Chinese: `r stat2.CN$sex_term`; UK: `r stat2.UK $sex_term`). These data support Prediction 2a, but not Prediction 2b.

Social Status (Prediction 3)

````
```{r}
stat3 <- apa_print(pred3, mse = FALSE, es = "pes")$full_result
```
````

Consistent with Prediction 3, Chinese women allocated significantly more mate dollars to social status for long-term relationships than did UK women (`r stat3$region`).

Creativity

````
```{r}
stat.creat.UK <- apa_print(creat.UK, mse = FALSE, es = "pes")$full_result
stat.creat.CN <- apa_print(creat.CN, mse = FALSE, es = "pes")$full_result
```
````

Analyses of creativity (Table 3) showed significant effects of relationship context in both samples (Chinese: `r stat.creat.CN$term`; UK: `r stat.creat.UK$term`) and a significant effect of participant sex in the Chinese sample (`r stat.creat.CN$sex`), but not the UK sample (`r stat.creat.UK$sex`). The interaction was not significant in either sample (Chinese: `r stat.creat.CN$sex_term`; UK: `r stat.creat.UK$sex_term`).

Kindness

````
```{r}
stat.kind.UK <- apa_print(kind.UK, mse = FALSE, es = "pes")$full_result
stat.kind.CN <- apa_print(kind.CN, mse = FALSE, es = "pes")$full_result
```
````

Analyses of kindness (Table 4) showed significant effects of relationship context in both samples (Chinese: `r stat.kind.CN$term`; UK: `r stat.kind.UK$term`) and a significant effect of participant sex in the UK sample (`r stat.kind.UK$sex`), but not the Chinese sample (`r stat.kind.CN $sex`). The interaction was significant in the UK sample (`r stat.kind.UK $sex_term`), but not the Chinese sample (`r stat.kind.CN$sex_term`).

Liveliness

````
```{r}
stat.lively.UK <- apa_print(lively.UK, mse = FALSE, es = "pes")$full_result
stat.lively.CN <- apa_print(lively.CN, mse = FALSE, es = "pes")$full_result
```
````

Analyses of liveliness (Table 5) showed significant effects of participant sex in both samples (Chinese: `r stat.lively.CN$sex`; UK: `r stat.lively.UK $sex`) and no significant effect of relationship context in either sample (Chinese: `r stat.lively.CN$term`; UK: `r stat.lively.UK$term`). The interaction was not significant in either sample (Chinese: `r stat.lively.CN$sex_term`; UK: `r stat.lively.UK$sex_term`).

Age

```{r}
stat.age.UK <- apa_print(age.UK, mse = FALSE, es = "pes")$full_result
stat.age.CN <- apa_print(age.CN, mse = FALSE, es = "pes")$full_result
```

In both samples, the main effects of participant sex (Chinese: `r stat.age.CN$sex`; UK: `r stat.age.UK$sex`) and relationship context (Chinese: `r stat.age.CN$term`; UK: `r stat.age.UK$term`) were significant. The interaction was significant in the Chinese sample (`r stat.age.CN$sex_term`), but not the UK sample (`r stat.age.UK$sex_term`). These results are summarized in Table 6.
